# Universal Health Coverage in Rural Ecuador: A Cross-sectional Study of Perceived Emergencies

**DOI:** 10.5811/westjem.2018.6.38410

**Published:** 2018-08-08

**Authors:** Martin Eckhardt, Dimitri Santillán, Tomas Faresjö, Birger C. Forsberg, Magnus Falk

**Affiliations:** * Linköping University, Department of Medical and Health Sciences, Division of Community Medicine, Linköping, Sweden; † Universidad Central del Ecuador, Facultad de Ciencias Médicas, Quito, Ecuador; ‡ Karolinska Institute, Department of Public Health Sciences, Stockholm, Sweden

## Abstract

**Introduction:**

In many low- and middle-income countries emergency care is provided anywhere in the health system; however, no studies to date have looked at which providers are chosen by patients with perceived emergencies. Ecuador has universal health coverage that includes emergency care. However, earlier research indicates that patients with emergencies tend to seek private care. Our primary research questions were these: What is the scope of perceived emergencies?; What is their nature?; and What is the related healthcare-seeking behavior? Secondary objectives were to study determinants of healthcare-seeking behavior, compare health expenditure with expenditure from the past ordinary illness, and measure the prevalence of catastrophic health expenditure related to perceived emergencies.

**Methods:**

We conducted a cross-sectional survey of 210 households in a rural region of northwestern Ecuador. The households were sampled with two-stage cluster sampling and represent an estimated 20% of the households in the region. We used two structured, pretested questionnaires. The first questionnaire collected demographic and economic household data, expenditure data on the past ordinary illness, and presented our definition of perceived emergency. The second recorded the number of emergency events, symptoms, further case description, healthcare-seeking behavior, and health expenditure, which was defined as being catastrophic when it exceeded 40% of a household’s ability to pay.

**Results:**

The response rate was 85% with a total of 74 reported emergency events during the past year (90/1,000 inhabitants). We further analyzed the most recent event in each household (n=54). Private, for-profit providers, including traditional healers, were chosen by 57.4% (95% confidence interval [CI] [44–71%]). Public providers treated one third of the cases. The mean health expenditure per event was $305.30 United States dollars (USD), compared to $135.80 USD for the past ordinary illnesses. Catastrophic health expenditure was found in 24.4% of households.

**Conclusion:**

Our findings suggest that the provision of free health services may not be sufficient to reach universal health coverage for patients with perceived emergencies. Changes in the organization of public emergency departments and improved financial protection for emergency patients may improve the situation.

## INTRODUCTION

Globally most deaths from injuries, infections, childhood diseases, and maternal conditions occur in low- and middle-income countries (LMIC). Many of these conditions can be effectively treated through immediate, inexpensive interventions; however, most LMIC still lack effective emergency medical systems to provide such interventions.^1^–^7^ In 2007 the World Health Assembly passed a resolution strengthening emergency care systems in LMIC, which led to increased interest and scientific literature related to the field.^4^,^5^,^7^–^9^

In many LMIC, emergency care is provided anywhere in the health system, including the primary care setting.^1^,^3^,^6^,^8^,^10^,^11^ For example, in Cuba emergency care is explicitly included in the primary care package,^12^ while many countries give it low priority.^1^ Most studies of medical emergencies are hospital based, while population-based investigations are scarce.^13^–^15^

A central element when studying emergency care is defining the term “medical emergency.” Health professionals usually define “emergency” as acute impaired physiology, and a threat to life, organs or limbs.^16^ According to Morgans and Burgess, a patient determines if he or she is experiencing an emergency or not based on layperson advice, psychosocial factors, and the pattern of the onset of symptoms.^17^

Poorer households in LMIC face barriers to emergency care due to weak or absent systems of financial protection.^1^ When faced with the choice to seek treatment or not these households choose between risking life or health and possible financial ruin.^1^,^3^ The latter is known as catastrophic health expenditure. It is widely defined as occurring when a household’s healthcare expenditure exceeds 40% of its ability to pay (ATP), i.e., their remaining income after basic needs are met.^18^,^19^

Latin American countries are moving closer towards universal health coverage (UHC), as endorsed by the United Nations and World Health Organization.^21^ UHC is part of the Sustainable Development Goals and is defined as everyone having access to needed health services without the risk of severe financial consequences.^22^ UHC was introduced in Ecuador in 2008 and includes emergency care.^20^,^23^ However, the country’s financing and delivery functions within the health system are still fragmented.^20^,^24^ The Ministry of Public Health (MPH) and the Ecuadorean Social Security Institute (Instituto Ecuatoriano de Seguridad Social, IESS) are the main public providers and run parallel systems of health centers and hospitals. The latter provides healthcare to entitled subscribing members.^25^ In addition, multiple private for-profit, non-profit, and traditional providers exist.^25^,^26^

López-Cevallos and Chi studied healthcare utilization in Ecuador using national data, showing that uninsured and rural dwellers have significantly lower odds of using hospital services.^25^ However, data on emergency care does not get presented separately. Guerra-Villavicencio’s analysis of national data from 2006–2014 reported an increased percentage of emergencies in the MPH health centers.^24^ Data concerning private providers do not exist. In an earlier, qualitative study conducted in rural Ecuador by our research group, we found indications that patients who perceive experiencing an emergency seek care from private providers.^27^

**Population Health Research Capsule**

What do we already know about this issue?

Ecuador has a universal health coverage (UHC) system providing all citizens the right to free healthcare in public facilities, including emergency care.

What was the research question?

What is the scope of perceived emergencies and the related healthcare-seeking behaviors in rural Ecuador?

What was the major finding of the study?

In the past year, 90/1,000 inhabitants experienced an emergency event. For-profit providers treated half of all cases.

How does this improve population health?

Free emergency care may not be sufficient to reach UHC for patients with perceived emergencies. Changes in public emergency departments may improve the situation.

The primary research questions for this population-based study, with the aim of exploring features of emergency healthcare-seeking in rural Ecuador, were the following: What is the scope of perceived emergencies?; What is the nature of perceived emergencies?; and What is the related healthcare-seeking behavior (HCSB)? Secondary objectives were to study the determinants of HCSB, compare the related health expenditure with expenditure from the past ordinary illness, and measure the prevalence of catastrophic health expenditure.

## METHODS

### Study Region

The study region is located in a rural rainforest area in Ecuador’s northwestern province Esmeraldas. Thirty communities are connected by muddy trails, usually traveled by foot or mule. The population size is approximately 5,000. Poverty is widespread and most households are dependent on subsistence farming or livestock breeding.^28^ A MPH health center operates in the central community, and traditional healers offer private services. There is a MPH hospital with a basic emergency department (ED), several private clinics, laboratories, pharmacies, and traditional healers located in the cantonal capital Quinindé. Private hospitals, specialized MPH hospitals and an IESS hospital are found in cities further away.

### Study Design

We conducted a cross-sectional explorative household survey during November–December 2012. The study unit was a household. We calculated the sample size using the formula:

n=N*X/(X+N-1)

where:

n = sample size
N = population size
*X =* [Z^2^_1-α/2_p(1–p)] / MOE^2^
Z^2^_1-α/2_*=* critical value of the Normal distribution a _1-α/2_ (for a 95% confidence level, α is 0.05 and Z^2^_1-α/2_ is 1.96)
p = the sample proportion
MOE = margin of error

Official census data was absent. Informal census data from 16 communities was revised with local key informants who had knowledge about recent migration. They also provided population estimates for the remaining communities. This resulted in the estimated number of households N=1,074. In 2011 the average household size was 4.76 people (Foundation Human Nature 2012, Informe del procesamiento y análisis estadístico de la información del censo: sector Y de la Laguna *[Information about data handling and statistical analysis of the census information: sector Y de la Laguna]*, Working Document, Foundation Human Nature Ecuador, Quito). Thus, the total population size was estimated to be 5,112.

For the primary outcome – the scope of perceived emergencies – we estimated the annual risk on the individual level at 10% (p = 0.1).^25^,^28^ The sample size was calculated with a 5% margin of error, 95% confidence interval (CI), and led to the conclusion that 135 people, representing 28 households, would have to be interviewed. The final step was to adjust for a response rate of 73% based on a previous study in the region, which resulted in 38 households.^28^

The calculations for one of the secondary outcomes, the prevalence of catastrophic household expenditure, were as follows. An individual’s risk of 10% to experience an emergency implies a 90% (0.9) risk of *not* experiencing an emergency. An average household’s annual risk to experience an emergency is therefore 1–(0.9^5^) = 0.409 (41%), yielding 440 households in the region. We assumed a 25% prevalence of catastrophic health expenditure, resulting in 110 households (10.2% of all households). With a 5% margin of error and 95% CI, this gave 123 households; adjustments for a 73% response rate resulted in a sample size of 168 households.

Due to the absence of exact population data, we applied two-stage cluster sampling with 30 clusters of seven households per cluster. Therefore, the probability of inclusion of a community was proportional to its size.^29^,^30^

### Questionnaires

We developed two, structured questionnaires using the World Health Survey 2002 as a reference.^31^ These were translated into Spanish by a bilingual speaker, pre-tested in the study region, and adjusted accordingly. Questionnaire 1 covered demographic and economic household data, expenditure data on the past ordinary illness, and the definition of perceived emergency. In the questionnaire, perceived emergency was defined as “a medical emergency exists, when a person has a health problem that you consider so urgent that you have to stop your current activity to seek help for this person (or yourself).” This definition was developed by two physicians and discussed and adjusted according to feedback from community health workers (CHWs) in the region. Questionnaire 2 covered the number of emergencies, symptoms, further case description, HCSB, and health expenditure. This questionnaire was administered directly after the first one when a household reported an emergency event.

### Variables Related to Primary Outcomes

We measured the scope of perceived emergencies by presenting the above definition and recording the number of events in the past year. The nature of the emergencies was assessed by recording symptoms and sorting these into chief complaints adapted to the rural Ecuadorean context.^33^,^34^ This was done independently by three physicians; discrepancies were discussed until consensus was reached. Further case description included perceived severity, hospitalization, surgery, days spent in bed, and decreased health status. Concerning HCSB, we collected the first provider contacted, reasons for this choice, and if a different choice would be made in case the emergency were to occur again. If several providers were contacted all were included.

For the analysis we constructed three dichotomous models, labeling cases either “public” or “private.” Model 1 represents the first contacted provider; the same applies to model 2 but here less serious cases were filtered out by excluding contact with traditional healers. (Based on local experience we assumed allopathic care is sought in more severe events.) In model 3, all contacted providers were aggregated. A case was labeled “public” if it was treated entirely within the public system (MPH, IESS) or “private” if at least one private provider was involved.

### Variables Related to Secondary Outcomes

The theoretical foundation to study determinants of HCSB is based on Andersen’s healthcare utilization model.^35^ The purpose is to discover which conditions facilitate or impede service utilization. In this model, the environment (healthcare system, external environment) and population characteristics (predisposing characteristics, enabling resources, need) influence HCSB (personal practices, service utilization), and outcomes (health status, satisfaction). Perceptions of severity are important determinants of HCSB.^36^ The entire system is dynamic with both individuals and systems learning from experience.^35^,^37^ Variables are displayed in Table 3. Our wealth index included eight typical household items (TV, DVD player, cellphone, refrigerator, motorbike, car, radio, and sound system) and was calculated using reciprocal proportions.^38^,^39^ Health expenditure data for past ordinary illness and emergency were collected. We excluded lost income due to the inability to work. As a proxy for household ATP we used reported household expenditure during the past month minus expenditure for food (including food produced by the household).^19^,^38^,^40^ This household expenditure was extrapolated over one year, and we calculated different thresholds of household ATP to determine if health expenditure was catastrophic.

### Interviewers, Interviewees, and Data Quality

Fifteen to 30 days prior to field research community leaders were informed via letter about aims and practical issues. Five CHWs were employed as interviewers, most of whom had not completed secondary school. To increase reliability, we chose to recruit four foreign medical students with good Spanish skills as observers.^32^ To maximize validity of the collected data, interviewers and observers were thoroughly trained by the first author during a five-day course. Most interviews took place in the interviewees’ homes, and some were held in a quiet spot following a community meeting.

The interview subjects were selected based on their seniority within the household and based on the occurrence of a perceived emergency during the past year. Heads of households (principal decision makers in the household) were interviewed with questionnaire 1. If absent, the next, most-senior person was interviewed. If at least one household member had experienced a perceived emergency in the past year, questionnaire 2 was used to interview the patient. If the patient was unavailable, under 15 years of age or had impaired memory about the event, the person who took care of the patient (caretaker) was interviewed. If both the patient and the caretaker were unavailable, then the head of the household was interviewed as a proxy respondent.^32^ The person who decided if and where to seek healthcare in the emergency situation was defined as “decision maker.” We included cases with ongoing treatment at the time of the study. If an eligible interviewee was not at home on two consecutive days the household was excluded and not replaced.

Observers were present at 70 interviews mainly at the beginning of the study and provided feedback to the interviewers.^32^ Questionnaires were checked by the first author at the end of each day. In case of missing or clearly erroneous data, interviewers revisited the households. Recall bias is related to less severe conditions that occurred a longer time ago.^41^ With our definition of perceived emergency we deemed a 12-month time period as manageable to keep recall bias to a minimum.^32^,^41^ Another source of error is familiarity of the interviewer with the respondent, thus tempting interviewers to help with the answers. To minimize this risk, we did not assign interviewers to their own community.

### Statistical Methods

All data were entered into a digital spreadsheet, double checked by two researchers, and transferred into SPSS (IBM Corp. SPSS Statistics for Macintosh, Version 23. Armonk, NY). We used descriptive statistics as outlined in the “Results” section. The CI was set at 95%. For 2x2 and 2x3 tables we used Fisher’s exact test.^40^ We did an independent samples T-test to compare means of health expenditures for private and public health contacts in model 3. Results were considered significant at p<0.05.

### Ethical Issues

Ethical approval was granted by the Bioethics Committee of the Pontificia Universidad Católica del Ecuador (Oficio-CBE-001-2013). The region’s “Farmers’ Health Committee” also approved the study. Participation was voluntary, with assured anonymity. We obtained written informed consent before the interviews, which could be interrupted at any time without negative consequences for the respondent.

## RESULTS

### General Results

**Figure 1:**
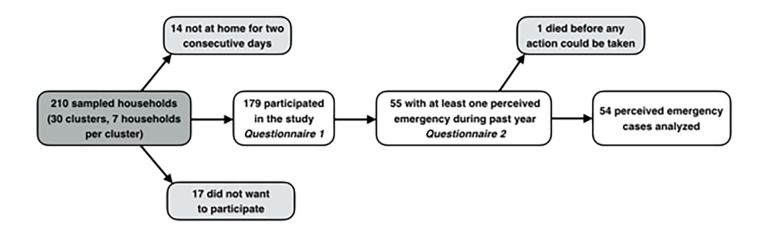
Sampled households, reasons for non-participation, and households interviewed with questionnaire 1 and 2.

The response rate was 85%. Figure 1 shows reasons for non-participation, and households interviewed with questionnaire 1 and 2. Characteristics of the interviewed households are shown in Table 1.

### Scope of Perceived Emergencies

Out of 179 households 55 had had at least one perceived emergency event during the past 12 months (30.7%; 95% CI [24.0–38.0%]). Several households reported more than one event; the total number of events was 74 (41.3%; 95% CI [34.0–49.0 %]). The mean number of events per affected household was 1.65 (95% CI [1.36–1.95]). The 179 participating households consisted of 825 persons, which yielded an incidence of 90 emergency events per 1,000 persons.

### Case Descriptions

One adult person died at home with the chief complaint of chest pain before any action could be taken, and therefore this case was excluded, except for case descriptions in Table 2. We further analyzed the perceived emergencies in the remaining 54 households. The majority of patients were 18–64 years old (58.2%), followed by the 5–17 age group (20.0%), under five years (18.2%), and the elderly (3.6%). Almost half of the cases (43.6%) were female. See Table 2 for case details. Public health insurance existed in 23.0% (95% CI [11–34%]) of households. Three households reported having private insurance. We found no statistically significant association between perceived severity and hospital admission. About 30% were admitted, one quarter of whom underwent surgery.

### Healthcare-seeking Behavior

**Figure 2:**
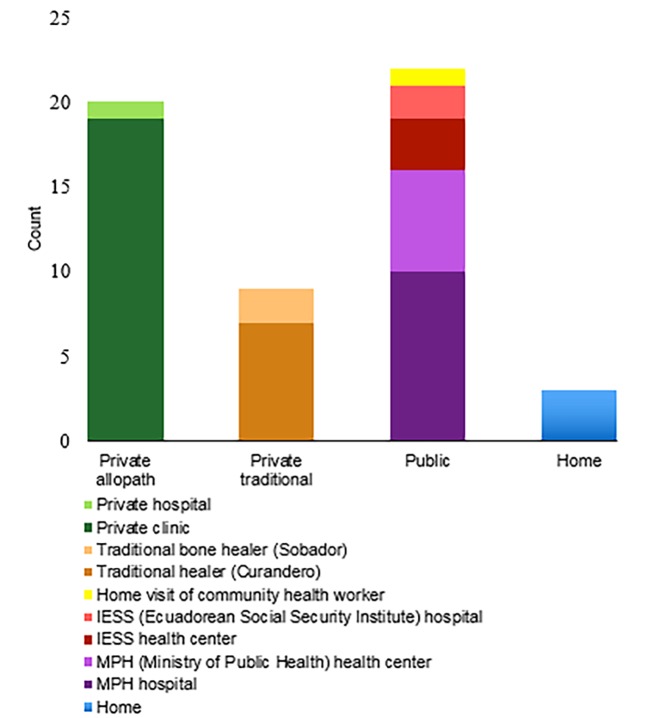
Initial management of the cases.

Figure 2 shows where cases were initially managed. Private providers were contacted in over half of the cases (57.4%; 95% CI [44–71%]). These providers were mainly clinics in the cantonal capital and cities farther away, followed by traditional healers outside the study region. Cases seen by traditional healers included a variety of conditions, from bites and other traumas to fever and seizures. Those who contacted a public provider mainly went to the MPH hospital in the cantonal capital or the MPH health center in the study region. Of the cases initially managed at home, one household lacked resources to travel with the patient at that point in time and two of the patients could not leave their homes due to weather conditions.

The upper part of Table 3 displays our models. Model 3 shows an aggregate of all contacted providers to treat a case. The number ranged from one to four (mean 1.5; 95% CI [1.3–1.7]). One-third of all households (n=18) cured their case entirely with public providers. All others had at least one private contact. Those who favored private allopathic and traditional care over the public (MPH, IESS) systems were interviewed about their reasons (n=31). The most frequent reasons given were as follows: difficulty to get seen by a public provider – including long wait times (32.3%); belief or trust in traditional medicine (29.0%); quick attention (22.6%); and trust in the chosen private allopathic provider (9.7%). Of those who had public health insurance 41.6% went to an IESS facility, while the rest sought private care. None sought treatment at a MPH facility. Concerning the question of whether different actions would be taken if the emergency were to occur again, 33.3% (n=18) of interviewees stated they would make a different choice. Of those, the majority (n=14) first had contact with a private provider (eight allopathic; six traditional). Those who saw an allopathic provider reported a range of different choices. Those who had visited a traditional healer showed a clear pattern to choose allopathic care if they were to experience the same emergency again.

### Determinants of Healthcare-seeking Behavior

Table 3 displays variables grouped into Andersen’s model and their influence on our utilization models 1–3. Due to the low number of observations, we applied wealth terciles. For the same reason, we grouped certain responses together such as cases perceived to be “life threatening” and “very serious”; regarding quality perception of the MPH system “bad” and “moderate” were grouped together. Statistically significant associations were found between seeking public care and membership in a community organization in models 1 and 3.

### Health Expenditure

The Ecuadorean currency is the United States dollar (USD). Total costs ranged from 0–6,000 USD, the median was $88.00 USD, and the mean $305.30 USD. In comparison, total costs for the past ordinary illness (n=143) were between $0–7,000 USD, with a median of $13.00 USD, and mean of $135.80 USD. Comparing expenditure means in model 3, we found “all public” to be $145.40 USD vs. $387.60 USD in the “at least one private provider” group. However, the difference was not statistically significant.

In perceived emergencies, the most expensive items were medicine and medical materials (mean $58.2 USD), followed by nonmedical costs for food and accommodation (mean $32.60 USD), transport (mean $22.20 USD), imaging studies (mean $12.70 USD), laboratory (mean $8.30 USD), consultation with an allopath (mean $6.00 USD), and consultation with a traditional healer (mean $5.40 USD).

### Catastrophic Health Expenditure

In the 41 cases for which expenditure data could be reliably collected, the mean household expenditure during the past month was $285.50 USD (95% CI [$204–366 USD]; median $213.50 USD). Table 4 displays the number and percentage of households exceeding different ATP cut-offs. Households at the 40% cut-off and above were analyzed further with regard to their healthcare utilization. Concerning utilization model 1, all but one household had contact with a private provider. In model 3, all households were in the “private” category. Due to the low number of observations, we did not analyze the determinants of catastrophic health expenditures (CHE).

## DISCUSSION

In this explorative study in rural Ecuador we found that perceived emergencies occurred in at least 30.7% of households per year, corresponding to 90/1,000 inhabitants. As most emergency studies are hospital based, this investigation provides important insights into the realities of emergencies for rural households. The most frequent chief complaints including fever, traumatic injury, and abdominal pain are in line with study results from a neighboring province, in which a well-functioning ED run with foreign aid was studied.^34^,^43^

The absence of an association between hospitalization and perceived severity by the decision maker suggests that they have difficulties in assessing the severity of a health condition. These difficulties have also been documented in a high-income country context.^17^ The percentage of hospital admissions is in line with results from the ED study mentioned above.^34^

Despite the national UHC policy, about half of all patients had their first contact with a private for-profit provider. The main reason for this was anticipated difficulties to be seen by a public provider. These difficulties include long wait times, possibly due to staff treating more severe cases. However, based on a study in Colombia other barriers for treatment in public EDs are likely to exist and would be worth investigating.^44^

Rather than being treated at the ED in the cantonal hospital most patients sought initial treatment at clinics and from traditional healers located in the same town or farther away, thus partly accepting longer travel distance. Others were taken to the nearby health center. These findings correspond with a study that reports low odds of use of hospital services for rural Ecuadoreans.^25^ As mentioned, this might be attributed to long ED wait times, which could be addressed with effective triage. If appropriate, cases could then be redirected to the appropriate provider, a system that works in a hospital in the neighboring province.^34^

An interesting finding concerning the first provider contact is that about one third of those who chose a private provider opted for a traditional healer, stating trust in traditional medicine as the reason. However, the majority of these respondents said they would choose allopathic care next time, indicating the learning process as described by Andersen.^35^

Analyzing determinants of HCSB, we found few statistically significant associations between our variables and public vs, private providers. This absence of statistically significant associations might be a reflection of the loss of power when performing sub-group analyses.

According to Andersen, services received for more serious health problems are primarily explained by need and demographic characteristics.^35^ Concerning perceived need, we found a trend between higher perceived severity and care seeking at private facilities. Comparing this to the reasons for seeking private care, a possible explanation may be that decision makers want to secure quick medical attention, and therefore disregard possible negative financial consequences. Membership in a community organization was significantly associated with seeking public care, likely indicating trust in public organizations.

About one quarter of all households had IESS public health insurance. An association between positive IESS status and use of IESS facilities could have been expected, but was surprisingly not found (models 1–3). Despite having insurance the majority of patients had contact with at least one private provider (model 3). This may indicate that the IESS does not provide services of the quality and timeliness that the decision makers desired. In fact, they were willing to pay despite the availability of free services. The timely provision of quality healthcare was what mattered to the population when choosing a service provider. Consequently, the free governmental services available to the study population did not properly protect households from out-of-pocket spending. This fact has also been reported in a CHE analysis across Latin America.^45^

We found high out-of-pocket expenditures for perceived emergencies compared to ordinary illness. The absence of a significant difference in expenditures between the public and private group in model 3 might depend on the weakness of the model. Nonmedical costs were the second and third most expensive items, which can be explained by the remote location of the study area and the number of contacted providers. Another study performed in a different rural Ecuadorean area, documented that the opening of a local hospital could substantially lower such costs.^46^ All but one of the households in the study that incurred CHE reported initial contact with a private provider, which suggests that private healthcare might contribute to the occurrence of CHE.

The prevalence of 24.4% of catastrophic health expenditure at the 40% ATP cut-off level and 31.7% at the 30% level is high. Knaul et al. report a prevalence of about 15% of CHE in rural Ecuador at the 30% cut-off level.^45^ However, this is not directly comparable as our results are only based on the past perceived emergency and exclude expenditures for other health problems. Nevertheless, our findings suggest that households that seek care for perceived emergencies face a high risk of financial catastrophe. Adequate financial protection for emergency patients is needed, especially for poor households.^1^

## LIMITATIONS

Due to the cross-sectional study design only self-reported outcomes and no clinical or longitudinal data were collected. Recall bias might have played a role regarding less severe cases, thus under-reporting is possible. Selection bias is believed to be minimal due to the 85% response rate, but not entirely absent. A possible confounder was households that had been established in the area within the 12-month recall period and reported emergencies from their time living elsewhere. However, other households may have moved away, thus we believe the impact on our results to be small. Our finding that many would not choose traditional healers again may be influenced by respondents wanting to please interviewers. Underestimation of total health expenditures is possible as some interviewees could not remember detailed cost information. Our proxy for ATP does not capture fluctuations of income over longer time periods; thus, over- and underestimations are possible. The calculation of CHE does not take loss of income due to inability to work into account. Furthermore, the calculation assumes that households facing high health expenditure can consume less essential goods, but leaves out coping mechanisms such as spending savings, selling assets etc.^45^ These shortcomings may have affected the results, but most likely not to the extent that our conclusions from the study are jeopardized.

## CONCLUSION

Perceived emergencies were found to be a frequent problem, occurring to 90/1,000 inhabitants in the past year. These events force the decision maker to quickly choose the “right” provider. In approximately half of all cases private for-profit providers were chosen. Health expenditure was found to be substantial even when compared to normal illness. The prevalence of catastrophic health expenditure was high. Our findings suggest that the provision of free health services may not be sufficient to reach UHC for patients with perceived emergencies. Changes in the organization of public EDs and improved financial protection for patients with emergencies may improve the situation. Further research should examine the options for financial protection in these conditions at a larger scale.

## Figures and Tables

**Table 1:** Characteristics of the interviewed households (n=179).

Household characteristic	Value	Comments
Mean number of household members (95% CI)	4.6 (4.3–4.9)	minimum-maximum: 1–12
Households with children <5 years	43.5%	
Households with members <18 years	77.7%	
Households with members >64 years	10.1%	
Households with members <18 and >64 years	82.1%	
Mean age of household head in years (95% CI)	44.0 (42.0–46.0)	3 missing
Number of household heads (%)		
Female	11 (6.1%)	
Male	168 (93.9%)	
Mestizo	175 (98.3%)	1 missing
Afro-Ecuadorean	3 (1.7%)	
No formal schooling & not completed primary school	76 (42.7%)	
Primary school completed & higher education	102 (57.3%)	1 missing
Marital status of the household head		
Living with partner	67.0%	
Married	24.6%	
Separated/divorced	4.5%	
Single	2.2%	
Widowed	1.7%	

*CI*, confidence interval.

**Table 2:** Description of the perceived emergency cases.

Chief complaint	Number	Perceived severity‡			Number of patients hospitalized	Number of hospitalized patients who had surgery	Average number of nights spent in hospital	Average number of days spent in bed outside hospital (all cases)	Number of patients with decreased state of health after the event

		Not very serious	Very serious	Life threatening					
Fever	21	6	11	4†	4	1	10.2	4.3	
Traumatic injury	10	2	6	2	3	1	2.3	21.3	4
Abdominal pain	5		4	1	1	1	3	11.2	1
Obstetrical complaint	3	1	2		3	1	3	13	
Chest pain	3	1	1	1*	1		1	6	1
Vomiting and/or diarrhea	2	1	1					2	
Convulsions/seizure	2		2		1		12	1	
Eye or ENT problem	1	1							
Weakness	1		1					5	
Vaginal bleeding, discharge, or breast complaint	1			1	1	1	3	8	
Upper or lower extremity complaint	1		no data					8	
Psychiatric/social problem	1		1					3	
Neurologic complaint	1			1				no data	1
Ingestion (accidental or intentional)	1			1	1		1	8	
Genitourinary problem	1		1		1		2	60	
Bites (human or animal)	1		1					2	

Total # (%)	55	12 (22.2%)	31 (57.4%)	11 (20.4%)	16 (29.6%**)	4 (25%)	4.94	7.2**	-

Empty cells = 0;

‡ data on one case missing;

† one patient had ambulatory surgery;

* person died before any action could be taken, excluded in the further presentation;

** the person who died is excluded from this calculation.

**Table 3:** Determinants of healthcare seeking behavior in perceived emergencies.

		Model 1 (first contacted provider)				Model 2 (first contacted provider)				Model 3 (all contacted providers to cure the case)		
		
		Public %	Private (allopathic & traditional) %	Statistics		Public %	Private (allopathic) %	Statistics		All public %	At least one private provider %	Statistics

All cases	n=54	42.6	57.4	95% CI for private: 44–71%	n=45	51.1	48.9	95% CI for private: 34–64%	n=54	33.3	66.7	95% CI for private: 54–80%

Variable				p value comments				p value comments				p value comments
Predisposing factors												
Patient age (<18, >18 years)	<18 years	39.1	38.7	1.000		39.1	31.8	0.758		44.4	36.1	0.569
Patient sex (female, male)	female	43.5	45.2	1.000		43.5	54.5	0.556		44.4	44.4	1.000
Sex of decision maker (female, male)	female	45.0	25.0	0.216 6 cases missing		45.0	31.6	0.514 6 cases missing		40.0	30.3	0.527 6 cases missing
Marital status of the household head	married	26.1	12.9	0.351		26.1	18.2	0.401		22.2	16.7	0.806
	living w/partner	69.6	74.2	0.351		69.6	63.6	0.401		72.2	72.2	0.806
	single*	4.3	12.9	0.351		4.3	18.2	0.401		5.6	11.1	0.806
Enabling factors												
Education of the household head (no formal schooling & primary not completed, primary completed & higher)	low	43.5	25.8	0.245		43.5	18.2	0.065		50.0	25.0	0.124
Education of the person who decided where to seek care (as above)	low	44.4	29.6	0.354 9 cases missing		44.4	22.2	0.289 9 cases missing		50.0	29.0	0.197 9 cases missing
Decision maker had experience with the type of emergency	yes	34.8	54.8	0.175		34.8	45.5	0.550		38.9	50.0	0.565
Patient having public health insurance	yes	21.7	24.1	1.000 2 cases missing		21.7	30.0	0.728 2 cases missing		16.7	26.5	0.507 2 cases missing
Household head member of a community organization	yes	34.8	9.6	0.039^†^		34.8	9.1	0.071		38.9	11.1	0.029^†^
Wealth index (terciles)	low	26.1	32.3	0.940		26.1	31.8	0.804		22.2	33.3	0.668
	middle	30.4	29.0	0.940		30.4	36.4	0.804		27.8	30.6	0.668
	high	43.5	38.7	0.940		43.5	31.8	0.804		50.0	36.1	0.668
Environmental factors												
Perceived quality of the MPH system (good, moderate/bad)	good	82.6	62.1	0.140 1 case missing		82.6	61.9	0.179 1 case missing		83.3	65.7	0.215 1 case missing
Seasons when the case occurred (rainy, dry)	rainy	26.1	35.5	0.560		26.1	31.8	0.749		33.3	30.6	1.000
Need factor												
Perceived severity of the case (not very serious, very serious/life threat)	very serious/life threat	63.6	87.1	0.055 1 case missing		63.6	90.9	0.069 1 case missing		64.7	83.3	0.167 1 case missing

Comments: Statistically significant values are ^†^ (p<0.05);

* widowed, separated, divorced, single.

*CI*, confidence interval; *MPH,* Ministry of Public Health; *w/,* with.

**Table 4:** Prevalence of catastrophic health expenditures (CHE), different cut-off levels.

Cut-off (of ability-to-pay)	Number of households	% of total households (n=41)
20%	15	36.6
30%	13	31.7
40%	10	24.4
50%	10	24.4
60%	10	24.4
70%	9	22.0
80%	8	19.5
